# X-linked adrenoleukodystrophy (X-ALD): clinical presentation and guidelines for diagnosis, follow-up and management

**DOI:** 10.1186/1750-1172-7-51

**Published:** 2012-08-13

**Authors:** Marc Engelen, Stephan Kemp, Marianne de Visser, Björn M van Geel, Ronald JA Wanders, Patrick Aubourg, Bwee Tien Poll-The

**Affiliations:** 1 Department of Neurology, Academic Medical Center, University of Amsterdam, Amsterdam, The Netherlands; 2 Department of Clinical Chemistry, Laboratory Genetic Metabolic Diseases, Academic Medical Center, University of Amsterdam, Amsterdam, The Netherlands; 3 Department of Pediatric Neurology/Emma Children’s Hospital, Academic Medical Center, University of Amsterdam, Amsterdam, The Netherlands; 4 Department of Neurology, Medical Center Alkmaar, Alkmaar, The Netherlands; 5 Department of Pediatric Neurology, Hospital Kremlin-Bicêtre, Assistance Publique des Hôpitaux de Paris, Paris, France; 6 INSERM UMR745, University Paris-Descartes, Paris, France

**Keywords:** X-linked adrenoleukodystrophy, X-ALD, Very long-chain fatty acids, VLCFA, ABCD1, Peroxisome, Myelin, Leukodystrophy, Demyelinating disorder, Addison’s disease, Myelopathy

## Abstract

X-linked adrenoleukodystrophy (X-ALD) is the most common peroxisomal disorder. The disease is caused by mutations in the *ABCD1* gene that encodes the peroxisomal membrane protein ALDP which is involved in the transmembrane transport of very long-chain fatty acids (VLCFA; ≥C22). A defect in ALDP results in elevated levels of VLCFA in plasma and tissues. The clinical spectrum in males with X-ALD ranges from isolated adrenocortical insufficiency and slowly progressive myelopathy to devastating cerebral demyelination. The majority of heterozygous females will develop symptoms by the age of 60 years. In individual patients the disease course remains unpredictable. This review focuses on the diagnosis and management of patients with X-ALD and provides a guideline for clinicians that encounter patients with this highly complex disorder.

## Disease names

X-linked adrenoleukodystrophy / adrenomyeloneuropathy / Schilder’s disease / sudanophilic leukodystrophy with melanoderma / encephalitis periaxialis diffusa / Siemerling-Creutzfeld disease.

## Definition

X-ALD is a metabolic disorder characterized by impaired peroxisomal beta-oxidation of very long-chain fatty acids (VLCFA; ≥ C22), which is reduced to about 30% of control levels [1,2]. Consequently, there is an accumulation of VLCFA in plasma and all tissues, including the white matter of the brain, the spinal cord and adrenal cortex. [3]. It is caused by mutations in the *ABCD1* gene located on the X-chromosome [4]. Mutations in this gene cause the absence or dysfunction of ALDP, a peroxisomal transmembrane protein that transports VLCFacyl-CoA esters from the cytosol into the peroxisome [5,6].

## Epidemiology

With an estimated birth incidence of 1 in 17,000 newborns (male and female) [7], X-ALD is the most common peroxisomal disorder. It occurs in all regions of the world [8]. Now that newborn screening has become technically feasible and may be implemented in some parts of the world [9], the true prevalence might be even higher.

## Clinical features of X-ALD

### A short history

In retrospect, the first cases of X-ALD were probably described in the late 19^th^ century. In 1897 Heubner described a young boy with rapidly progressive neurologic deterioration consistent with X-ALD, classified as having “diffuse sclerosis” on autopsy [10]. This designation was used at the time for any disease of the white matter causing hardening of the tissue. Cases of “diffuse sclerosis” that resemble X-ALD were also described in 1899 by Ceni [11] and in 1910 by Haberfield and Spieler [12]. Shortly thereafter Schilder suggested that “diffuse sclerosis” was too vague and proposed a more accurate pathological classification of the leukodystrophies [13-15]. He described several cases with lesions in the cerebral white matter and perivascular inflammation that he named “encephalitis periaxialis diffusa”. The syndrome of rapidly progressive cerebral demyelination with inflammatory changes in the white matter on autopsy became known as “Schilder’s disease”. Many cases were described in the following fifty years [16]. Nevertheless, Siemerling and Creutzfeldt are often credited for having described the first case of X-ALD because they were the first to describe the association of acute cerebral demyelination with clinical and pathological signs of Addison’s disease [17].

X-ALD has remained an enigmatic disease for most of the 20^th^ century, although some suspected it might be a metabolic disorder affecting both the central nervous system and adrenal glands [18,19]. Based on the presence of lipid inclusions in adrenal glands, testis, brain and Schwann cells, Schaumburg and Powers suggested the term adrenoleukodystrophy for the disorder and speculated it might be a lipid storage disorder [20]. This hypothesis was confirmed when post-mortem biochemical analysis of brain, adrenal glands and serum of seven patients revealed the presence of cholesterol esters with high amounts of fatty acids longer than C22. These fatty acids were referred to as very long-chain fatty acids (VLCFA) [21]. X-linked adrenoleukodystrophy now became a distinct clinical entity with an associated biochemical marker that could be used to confirm the diagnosis in readily accessible materials like blood cells and plasma [3].

Already in 1910 Addison’s disease associated with spastic paraplegia was described [22]. Familial Addison disease with or without spastic paraplegia was usually considered to be a variant of hereditary spastic paraplegia [23,24]. In 1976, an X-linked adult onset progressive myelopathy that was often associated with Addison’s disease was reported [25]. A year later, five more cases were described and the term adrenomyeloneuropathy (AMN) was proposed because of the involvement of the adrenal cortex, spinal cord and peripheral nerves [26]. With plasma VLCFA analysis as newly identified biomarker, AMN was soon found to be a form of X-ALD thus extending the phenotypic spectrum of X-ALD [3]. Now, we classify the symptomatology of X-ALD in children and adults in several phenotypes Table 1. They will be discussed here in more detail. 

**Table 1:** The X-ALD phenotypes

	**CCALD**	**AdolCALD**	**ACALD**	**AMN**		**Addison only**	**Women with X-ALD**
				**No cerebral disease**	**Cerebral disease**		
Frequency (%)	31 - 35	4 - 7	2 - 5	40 - 46	20% of AMN patients over a period of 10 years	Decreasing with age	unknown how many are symptomatic
Age at onset	2.5 - 10	10 - 21	> 21	> 18	> 18	> 2	highly variable, mostly > 40
Myelopathy	-	Possible at a preclinical stage	+ or -	+	+	-	+
White matter lesions on brain MRI	Extensive	Extensive	Extensive	Wallerian degeneration of the	parieto-occipital, frontal, or	-	Very rare
				corticospinal tracts in brain-stem, pons and internal capsules	involving the centrum semiovale		Wallerian degeneration of the corticospinal tracts in brain-stem, pons and internal capsules is less common than in males with AMN
Behavioral and cognitive disorder	+	+	+	-	+	-	Very rare
Peripheral neuropathy	-	rare	possible	Sensory-motor, mostly axonal, rarely demyelinating	Sensory-motor, mostly axonal	-	+/−
Endocrine disorder	often AD	often AD	often AD	often AD and testicular insufficiency	often AD and testicular insufficiency	AD	AD rare (< 1%)
Progression	rapid	rapid	rapid	slow	rapid	-	slow

- = absent; + = present; CCALD = childhood cerebral ALD; AdolCALD = adolescent cerebral ALD; ACALD = adult cerebral ALD; AMN = adrenomyeloneuropathy; AD = Addison-disease.

## Phenotypes in male X-ALD patients

### Addison-only

Adrenocortical insufficiency (or even an Addisonian crisis) can be the presenting symptom of X-ALD in boys and men, years or even decades before the onset of neurological symptoms. X-ALD is a frequent cause of Addison’s disease in boys and adult males [27,28], in particular when circulating adrenocortical autoantibodies are absent [29]. A percentage as high as 35 has been reported [27]. However, this figure may be overestimated. Indeed, in a study of 40 Dutch men with adrenocortical insufficiency, no patients with X-ALD were identified (B.M. van Geel BM and J. Assies unpublished data). Recognizing that Addison’s disease is due to X-ALD has important implications for genetic counseling but also management. It is therefore important to consider X-ALD in any boy or adult male presenting with Addison’s disease. Adrenocortical insufficiency initially affects the glucocorticoid function of the adrenal, but the mineralocorticoid function is ultimately deficient in approximately half of the X-ALD patients [27].

### Cerebral ALD (childhood, adolescent and adult)

These phenotypes are the most rapidly progressive and devastating phenotypes of X-ALD. They most frequently present in childhood (childhood cerebral ALD; CCALD), however never before the age of 2.5 years [30,31]. The onset of CCALD is insidious, with deficits in cognitive abilities that involve visuospatial and visuomotor functions or attention and reasoning. In boys and adolescents it initially results in a decline of school performance. These early clinical symptoms are often misdiagnosed as attention deficit hyperactivity disorder and can delay the diagnosis of CCALD. As the disease progresses, more overt neurologic deficits become apparent, which include withdrawn or hyperactive behavior, apraxia, astereognosia, auditory impairment ("word deafness'' reflecting impairment in acoustic analysis of word sounds), decreased visual acuity, hemiparesis or spastic tetraparesis, cerebellar ataxia and seizures. At this stage progression is extremely rapid and devastating. Affected boys can lose the ability to understand language and walk within a few weeks. Eventually, patients are bedridden, blind, unable to speak or respond, requiring full-time nursing care and feeding by nasogastric tube or gastrostomy. Usually death occurs two to four years after onset of symptoms, or - if well-cared for - patients may remain in this apparent vegetative state for several years [30].

**Figure 1:**
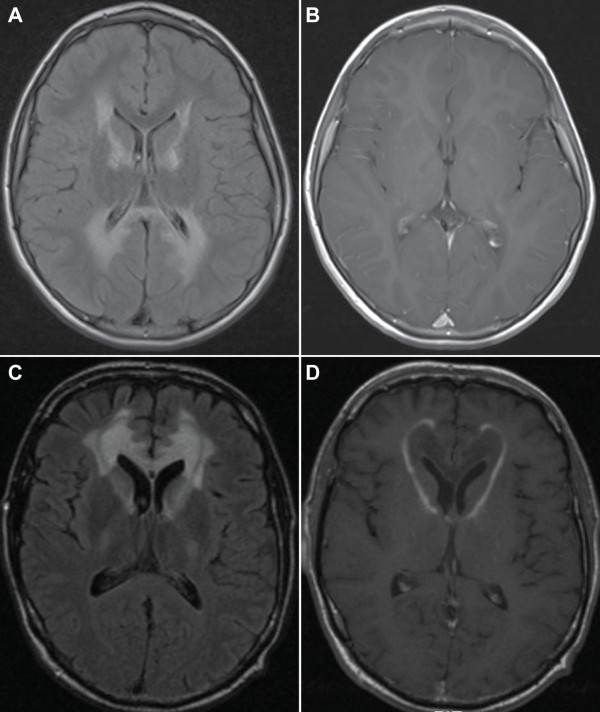
**MRI of the brain in a case of childhood cerebral ALD showing characteristic extensive white matter changes in the parieto-occipital region and internal capsules on FLAIR sequences (A).** This area is initially affected in about 80% of cases of cerebral ALD. The rim enhances after administration of gadolinium on T1 sequences (**B**). In about 20% of cases the site of initial involvement in cerebral ALD is the frontal white matter as shown on this FLAIR image of a different patient with cerebral ALD (**C**), with prominent rim enhancement after administration of gadolinium on a T1 weighted image (**D**).

The rapid neurologic decline of CCALD is caused by a severe inflammatory demyelination process primarily affecting the cerebral hemispheres. Postmortem histopathological examination of brain tissue reveals extensive demyelination with perivascular infiltration of lymphocytes and macrophages that resembles, to some extent, the demyelinating lesions seen in multiple sclerosis [32]. In 80% of patients the initial demyelinating lesion is localized in the splenium of the corpus callosum and progresses to involve the adjacent parieto-occipital white matter [33]. Alternatively, the initial demyelinating lesions may occur in the genu of corpus callosum and then progress symmetrically or asymmetrically to the white matter of the frontal lobes [33]. The lesions may also initially involve the pyramidal tracts within the pons or the internal capsules and then extend into the white matter of the centrum semiovale. Brain MRI shows abnormal signal intensities (increased signal on T2 and FLAIR sequences, decreased signal on the T1 sequence) in the corpus callosum, parieto-occipital or frontal white matter or pyramidal tracts within the brainstem, pons and internal capsules (Figure 1). Atypical patterns of demyelination, for instance highly asymmetrical disease have been described [34]. Initially, the demyelinating lesions do not show enhancement on T1 sequences after intravenous gadolinium administration. The presence of gadolinium enhancement of demyelinating lesions occurs usually in a second stage when the disease starts to progress rapidly, reflecting severe inflammation and disruption of the blood brain barrier. This transition to the rapidly progressive neuroinflammatory stage may occur very early, even when the demyelinating lesions are restricted to the corpus callosum or pyramidal tracts or later, once the demyelinating lesions have already significantly extended into the cerebral white matter. In the absence of biomarkers to predict this evolution, brain MRI remains the only tool to detect this evolution in an early stage. A scoring system to grade the demyelinating abnormalities on brain MRI has been developed by Loes [35]. This score correlates well with neurologic symptoms when the demyelinating lesions involve the parieto-occipital white matter. However, lesions in the frontal white matter can produce severe symptoms (especially behavioral), while the Loes score is still low. Less frequently, cerebral demyelination as the presenting phenotype of X-ALD occurs in adolescence (AdolCALD) or adulthood (ACALD). The symptomatology in these patients strongly resembles CCALD, but the initial progression of symptoms usually is slower. In adults, the early cognitive decline is rarely recognized by their families and friends or at work. As the disease progresses, psychiatric disturbances mimicking schizophrenia or psychosis are not uncommon [36]. In those cases, the diagnosis of X-ALD is often delayed; particularly when no family history of X-ALD is present and when clinical symptoms of Addison's disease are absent. 

Approximately 10% of boys or adolescents with cerebral ALD may not develop the rapidly progressive neuroinflammatory stage of the disease. The same may occur in men with ACALD or in men with AMN who develop secondary cerebral demyelination (see below). This cerebral demyelinating form of X-ALD is often referred to as “chronic or arrested cerebral X-ALD”. The cerebral demyelinating process arrests spontaneously and the patient can remain stable for a decade or even longer. But even after a 10–15 year period of stability, sudden onset of rapid neurologic deterioration may occur, reflecting a full progression to the neuroinflammatory stage of the disease.

### Adrenomyeloneuropathy (AMN)

Virtually all patients with X-ALD who reach adulthood develop AMN, usually in the 3^rd^ and 4^th^ decade. Initial symptoms are limited to the spinal cord and peripheral nerves. Patients develop gradually progressive spastic paraparesis, sensory ataxia with impaired vibration sense, sphincter dysfunction (mostly urinary), pain in the legs and impotence [37]. The clinical burden of peripheral nerve involvement is usually moderate and difficult to assess because of prominent spinal cord symptoms. If polyneuropathy is investigated electrophysiologically, an axonopathy is found in the majority of the patients [38]. Rarely, the initial symptomatology may be that of a peripheral neuropathy, either demyelinating or axonal [39,40].

**Figure 2:**
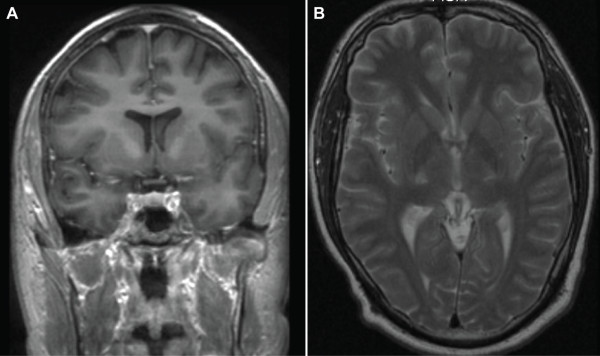
MRI of the brain in a patient with AMN showing increased signal in the pyramidal tracts on T2-weighed coronal (A) and axial (B) images indicative of Wallerian degeneration.

**Figure 3:**
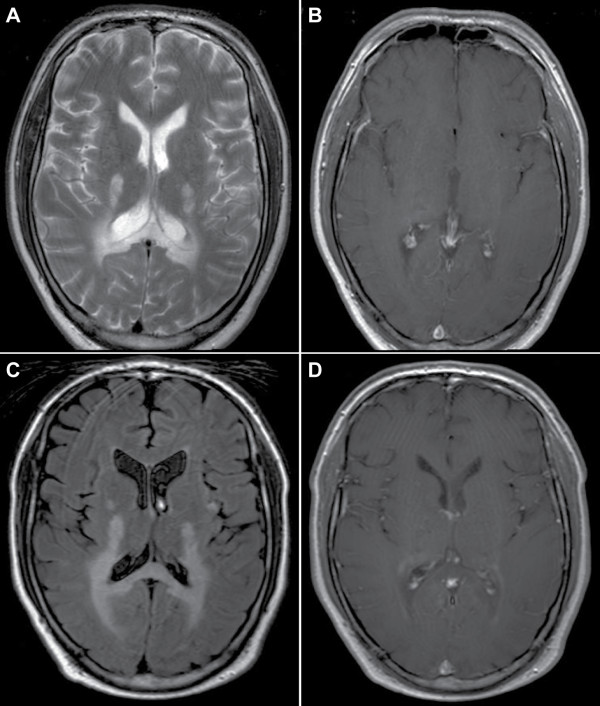
**MRI of the brain (T2 (A) and FLAIR (C) images; T1 with gadolinium (B, D)) of a patient with AMN who rapidly deteriorated clinically with new symptoms of cognitive decline.** On MRI extensive white matter changes were seen in the parieto-occipital white matter and corpus callosum **(A)**, but no enhancement of the lesion after administration of gadolinium **(B)**. A follow-up MRI about 3 months later shows progression of the white matter lesion **(C)** and there is now faint enhancement of the rim of the lesion after gadolinium administration **(D).**

Before the availability of MR imaging, AMN was often misdiagnosed as multiple sclerosis or hereditary spastic paraparesis (HSP). The pathological basis of AMN is a noninflammatory distal axonopathy that involves the long tracts of the spinal cord and, to a lesser extent, the peripheral nerves [41]. This phenotype is in most cases slowly progressive, causing severe motor disability of the lower limbs over one or two decades but mild or no significant deficits in arms and hands. Brain MRI is normal or may show subtle abnormalities such as moderately increased signal intensities of the pyramidal tracts in brainstem, pons and internal capsules on FLAIR and T2 sequences that reflect likely Wallerian degeneration in patients with longstanding symptoms of AMN (Figure 2). These abnormalities are not considered manifestations of cerebral ALD. However, if the increased signal of the pyramidal tracts becomes more intense and extends beyond the internal capsules into the white matter of the centrum semiovale, this is considered a manifestations of cerebral ALD (Figure 3). MRI of the spinal cord eventually shows non-specific atrophy, but no demyelination or gadolinium enhancement as seen in multiple sclerosis. In contrast, magnetization transfer or diffusion tensor-based imaging sequences show significant abnormalities of the spinal cord tracts [42-44]. 

**Figure 4:**
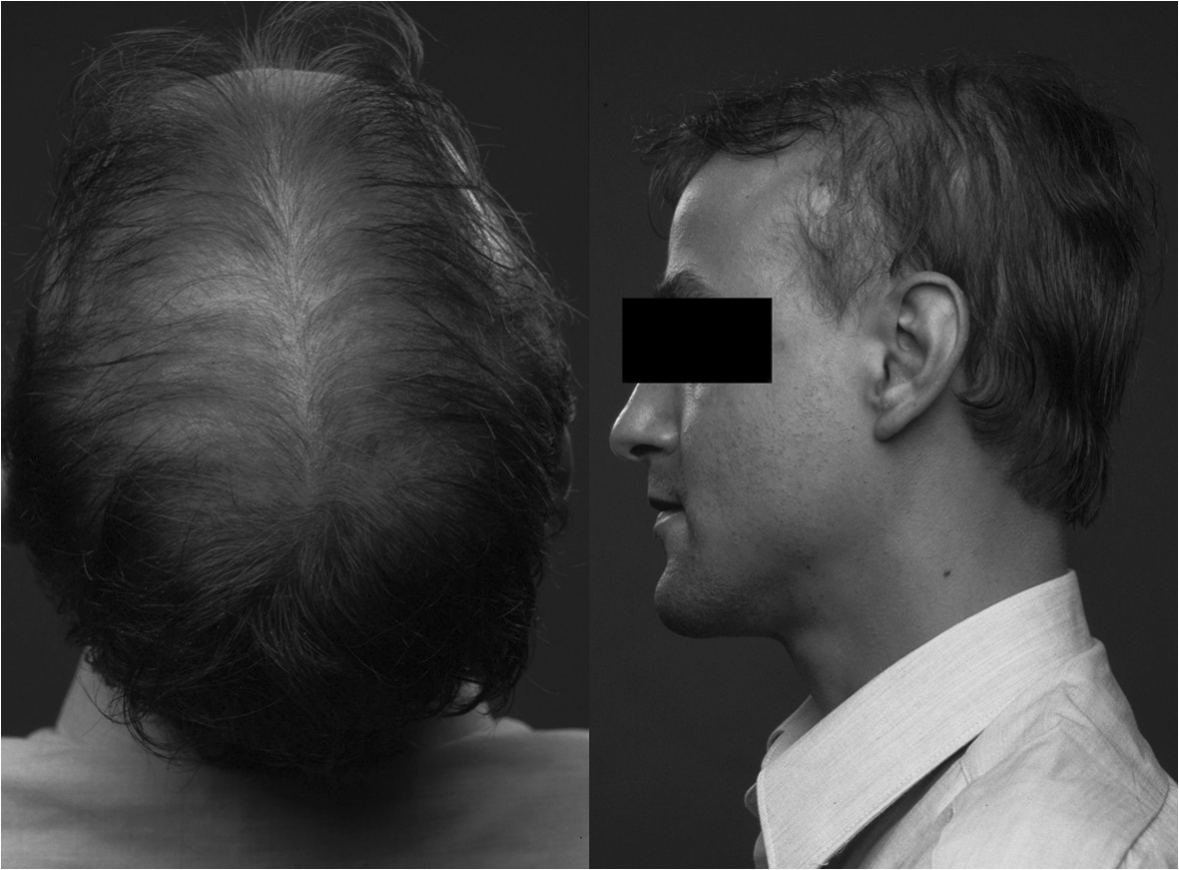
**Thin and scanty scalp hair in a man with X-ALD (AMN phenotype).** Written informed consent was obtained from the patient for publication of these images.

A retrospective study revealed that over a period of 10 years, approximately 20% of AMN patients developed additional cerebral demyelination [45]. After an initial progression demyelinating lesions can stabilize spontaneously leading to moderate cognitive deficits. However, once the cerebral demyelinating lesions have entered the active phase of neuroinflammation with gadolinium enhancement, the prognosis is as poor as in CCALD. The neurologic symptoms of AMN patients that develop cerebral ALD are identical to those observed in adults with adult cerebral ALD, with additional symptoms of the pre-existing myelopathy. Approximately 70% of AMN patients have adrenocortical insufficiency [46]. An equal percentage of affected males have subclinical signs of testicular insufficiency [47]. Clinical symptoms of testicular insufficiency are rare. Hair of patients with AMN is often thin and sparse; these patients often show balding at an early age (Figure 4). This typical scanty scalp hair was first described in 1955 [23]. 

### Women with X-ALD

As in many X-linked diseases, it was assumed that female carriers remain asymptomatic. However, many women develop AMN-like symptoms [48]. Physical examination of a large group of female carriers attending family conferences in the United States revealed that more than 50% had some kind of abnormality on neurologic examination [30]. An increasing number of symptomatic heterozygous women are identified as the first member of their family to be affected by X-ALD and the real incidence of AMN in heterozygous women is likely to be close to 65% by the age of 60 years (Engelen et al, manuscript in preparation). Onset of neurologic symptoms mainly occurs between the 4^th^ and 5^th^ decade and they are very similar to those observed in adult males with AMN. Sensory ataxia, fecal incontinence and pain in the legs are however often more prominent in symptomatic women with AMN. Cerebral involvement and adrenocortical insufficiency are rare, 2% and 1%, respectively [30,49]. So far, neither have been documented in the Netherlands and France. It is speculated that skewed X-inactivation in neuronal cells may contribute to the manifestation of neurologic symptoms in X-ALD carriers [50,51]. In a recent study the association of skewed X-inactivation and symptomatic status could not be confirmed [52]. However, this may be attributable to differences in mean age between the symptomatic and asymptomatic group in this study.

### Asymptomatic and pre-symptomatic patients

A diagnosis of X-ALD must be followed by extended family screening together with a geneticist. This enables the detection of: 1) heterozygous women who can be offered prenatal or in some countries preimplantation diagnosis for future pregnancies, and 2) boys or adult males who are asymptomatic but at risk to develop cerebral demyelination or adrenocortical insufficiency [8]. It is important to detect these complications as early as possible for treatment, as described in the section on ‘clinical management’.

### Evolution over time

X-ALD phenotypes are not static. Presymptomatic males are nearly all at risk to develop neurologic (cerebral ALD, AMN) or endocrinologic (Adisson’s disease) symptoms. Addison-only males can develop AMN or cerebral ALD and AMN males can develop cerebral demyelination. It is estimated that over a period of 10 years about 20% of patients with AMN will progress to a cerebral phenotype [45]. Presymptomatic women with X-ALD are mostly at risk to develop AMN. Progression of X-ALD in a specific individual can not be predicted.

X-ALD is characterized by the absence of a genotype-phenotype correlation. In spite of identical *ABCD1* gene mutations, patients can have markedly divergent neurological and neuropathologic characteristics [53,54]. There is ample evidence that other genetic and/or environmental factors may influence the clinical presentation of X-ALD. Segregation analysis suggests that the phenotypic variability is due to at least one autosomal modifier gene [55,56]. Head trauma may be an environmental factor triggering the onset of cerebral ALD [57]. Other brain lesions, like a stroke, may also trigger cerebral demyelination in patients with X-ALD.

## Etiology and pathophysiology

X-ALD is caused by mutations in the *ABCD1* gene located on the X-chromosome. So far, 600 different mutations have been identified (see http://www.x-ald.nl). *ABCD1* encodes ALDP, a peroxisomal transmembrane protein involved in the transport of VLCFA-CoA esters from the cytosol into the peroxisome [5,6,58]. ALDP deficiency leads to impaired VLCFA beta-oxidation (about 30% of normal) and the accumulation of VLCFA-CoA esters in cells. The VLCFacyl-CoA esters in the cytosol are incorporated into various lipid fractions and are also substrate for further elongation to even longer fatty acids [6,59]. ELOVL1 (elongation of very-long-chain-fatty acids) is the single elongase catalyzing the synthesis of both saturated VLCFA (C26:0) and mono-unsaturated VLCFA (C26:1). ELOVL1 expression is not increased in X-ALD fibroblasts, but increased synthesis of VLCFA is due to increased substrate availability [6].

Various *in vitro* experiments have demonstrated that VLCFA accumulation is toxic. VLCFA are extremely hydrophobic and their rate of desorption from biological membranes is about 10,000 times slower than that of long-chain fatty acids causing disruptive effects on the structure, stability and function of cell membranes [60,61]. Excess of VLCFA in cultured cells decreases the ACTH-stimulated cortisol release by human adrenocortical cells [62] and causes cell death in astrocytes and oligodendrocytes [63]. *In vivo*, VLCFA cause oxidative stress and oxidative damage to proteins [64,65] microglial activation and apoptosis [66]. VLCFA-induced oxidative stress may contribute to axonal damage in the spinal cord of *Abcd1* knock-out mice that develop an AMN-like phenotype [64,67]. In addition, a dysregulation of oxidative phosphorylation, adipocytokine and insulin signaling pathways, and protein synthesis was recently shown in the spinal cord of *Abcd1* knock-out mice [68].

The neuropathological hallmark of AMN is an axonopathy with microgliosis but without significant myelin changes. It is therefore likely that the primary consequence of VLCFA accumulation impairs the capacity of oligodendrocytes and Schwann cells to sustain axonal integrity, resulting in axonal damage.

The VLCFA homeostasis in X-ALD is disturbed [59]. This may contribute to the destabilization of the myelin sheath and impair the function of astrocytes and microglia which play an important role in myelin integrity [63,66]. Not all males with X-ALD develop cerebral ALD corroborating the notion that additional triggers, genetic, epigenetic and/or environmental, modify this process.

In the absence of a relevant X-ALD mouse model that develops cerebral demyelination with neuroinflammation, the pathogenic processes that result in cerebral demyelination and subsequently severe neuroinflammation remains therefore poorly understood.

## Biochemical and molecular diagnosis

Newborn screening is now technically feasible [9]. It is based on the measurement of C26:0 lysophosphatidylcholine (26:0-lyso-PC) in dried blood spots. It will lead to identification of pre-symptomatic patients with X-ALD. Whether this screening is implemented is a matter of national policy and dependent on ethical considerations.

If X-ALD is suspected in a male with neurological symptoms (with or without typical brain MRI abnormalities) or Addison’s disease, demonstration that VLCFA are elevated in plasma confirms the diagnosis. For women with X-ALD, the diagnostic test of choice is mutation analysis of the *ABCD1* gene, because 15% of women with X-ALD have normal plasma VLCFA levels [37]. Family screening follows the same recommendations.

The *ABCD1* gene is the single causative gene for X-ALD [4]. It is an X-linked inherited disorder. Therefore all daughters of an affected male are obligate carriers whereas his sons can never be affected. When a woman carries the gene for X-ALD, there is a 50% probability for each pregnancy that the gene is transmitted to a son or daughter. The frequency of *de novo* mutations in the index case is estimated to be around 4% [69] which indicates that the *ABCD1* mutation occurred in the germ line. There is evidence of gonadal or gonosomal mosaicism in less than 1% of patients which means an increased risk of an additional affected offspring.

## Differential diagnosis

### Symptoms

In boys and men presenting with Addison’s disease, including those who have only a glucocorticoid deficiency, and in whom there are no detectable steroid-21-hydroxylase or other organ-specific antibodies, X-ALD should be considered and determination of plasma VLCFA levels must be performed [70,71]. Young boys and adult males presenting with cognitive and neurological symptoms with (usually enhancing) white matter lesions on brain MRI should be tested for X-ALD.

In adult men, the most common presenting symptom of X-ALD is a chronic myelopathy. In the past, AMN was often misdiagnosed as primary progressive multiple sclerosis or hereditary spastic paraparesis. After ruling out a compressive myelopathy by MRI of the spinal cord and other common causes of chronic myelopathy (some possible diagnoses for chronic myelopathy with a (near) normal MRI are summarized) [72], X-ALD should be considered. A clinical clue to the diagnosis can be the presence of adrenocortical insufficiency and early baldness. However, even in the absence of clinical signs of adrenocortical insufficiency AMN should be considered.

Differential diagnosis of chronic myelopathy with normal MRI:

Deficiencies: Vitamin B12, folic acid, copper

Hereditary spastic paraparesis with amyotrophy

Infections (e.g., HTLV-1, HIV)

Primary lateral sclerosis

Radiation myelopathy

Cerebrotendinous xanthomatosis

Metachromatic leukodystrophy

Krabbe disease

A category that remains underdiagnosed relatively often are women with AMN. Physicians familiar with X-ALD are aware that it is not uncommon that women with X-ALD develop a myelopathy [46]. However, most neurology textbooks do not list X-ALD in the differential diagnosis for chronic myelopathy in women. The diagnosis can only be excluded by *ABCD1* mutation analysis. As mentioned before, there is an increasing number of X-ALD families in which the index case is a woman with clinical symptoms of AMN.

### Increased VLCFA

Increased plasma VLCFA are not pathognomonic for X-ALD or might even be false positive, because of hemolysis of the sample or dietary causes, for example a ketogenic diet [73]. Alternatively, some dietary products like rapeseed oil or mustard seed oil that are rich in erucic acid (C22:1) can result in the lowering of C26:0 therefore causing a false negative result (Ann Moser, personal communication). If metabolic screening reveals increased VLCFA, the next step is to confirm the diagnosis by performing *ABCD1* mutation analysis. If this is negative, it is important to consider other peroxisomal disorders, such as 1) peroxisomal biogenesis disorders with late onset of symptoms [74,75] (Ebberink et al, *in press*), and 2) peroxisomal acyl-CoA oxidase 1 (ACOX1) or D-bifunctional enzyme (DBP) deficiency with late onset of symptoms. This requires further testing for bile acid intermediates, phytanic and pristanic acids in plasma, and plasmalogens in erythrocytes [76]. A further metabolic screening of peroxisomal dysfunction must also be performed on skin fibroblasts, at least by performing catalase immunofluorescence after culturing cells at 37°C, but also at 40°C [77].

### White matter changes on MRI

In males with confluent white matter changes, X-ALD should be considered, especially when there is increased signal intensity on T2-weighed and FLAIR sequences in the parieto-occipital region and the splenium of the corpus callosum [33]. Rim enhancement of demyelinating lesions is usually observed when boys or adult males present with overt neurological symptoms from cerebral ALD. Therefore, intravenous administration of gadolinium is advised if X-ALD is considered. However, in about 20% of males with X-ALD, the white matter changes occur predominantly in the genu of corpus callosum and frontal lobes, or involve the pyramidal tracts with extension in the white matter of the centrum semiovale [33]. The brain MRI pattern of cerebral X-ALD is usually readily recognized by neuroradiologists. A helpful algorithm for diagnostic work-up of patients with white matter changes on brain MRI is suggested by Schiffmann and Van der Knaap [78].

There are other peroxisomal disorders that present with MRI abnormalities resembling X-ALD, in particular peroxisomal biogenesis disorders and ACOX1 or DBP deficiency with late onset of symptom [79]. Although the radiologic findings might suggest X-ALD, the clinical presentation is very different and therefore these disorders are easily differentiated.

## Genetic counseling and prenatal diagnosis

Genetic counseling must be offered to the parents of affected boys, adult males and women with X-ALD and their family to detect: 1) carriers who can be offered prenatal diagnosis, and 2) asymptomatic or pre-symptomatic men or women who can benefit from therapeutic interventions. Regular follow-up in presymptomatic males can prevent serious morbidity and mortality.

*ABCD1* mutational analysis can be performed either on a fresh chorionic villus sample at 11–13 weeks of pregnancy or on amniotic cells obtained from amniotic fluid after centrifugation at 15–18 weeks of gestation [80]. In some countries, pre-implantation genetic diagnosis is available. If the fetus is a female, there is no consensus with respect to prenatal diagnosis and termination of pregnancy, due to the highly variable expression of disease in women with X-ALD. Cases will be evaluated on an individual basis.

## Clinical management

**Figure 5:**
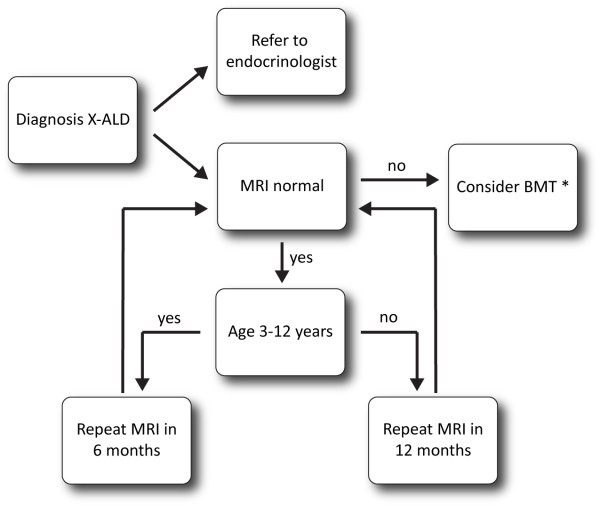
**Flowchart describing the outpatient management of X-ALD.** *If there is no gadolinium enhancement present, consider arrested cerebral ALD and repeat the MRI in 3 months.

The flowchart in Figure 5 summarizes the recommendations for follow-up of boys and men with X-ALD.

### Boys and adult males with X-ALD

Follow-up in boys and men with X-ALD is important for two reasons: 1) early detection of adrenocortical insufficiency and 2) early detection of cerebral ALD to propose allogeneic hematopoietic stem cell transplantation (HCT) if a HLA-matched donor or cord blood is available. Despite significant mortality risk, allogeneic HCT remains the only therapeutic intervention that can arrest the progression of cerebral demyelination in X-ALD, provided the procedure is performed very early, i.e., when affected boys or men have no or minor symptoms due to cerebral demyelinating disease [81,82].

In the future, transplantation of autologous hematopoietic stem cells that have been genetically corrected with a lentiviral vector before re-infusion might become an alternative to autologous HCT, once the very encouraging results obtained in the first two treated patients will have been extended to a larger number of patients with cerebral X-ALD [83].

If boys or men do not have Addison’s disease it is recommended that they are evaluated yearly by an endocrinologist for adrenocortical dysfunction by measuring the plasma ACTH levels and performing an ACTH stimulation test [71]. Steroid replacement therapy can then be initiated if necessary.

Boys without neurological deficits should be monitored closely for radiological signs of cerebral ALD. CCALD has not been reported before the age of 2.5 years [8]. We recommend an MRI of the brain every 6 months in boys aged 3 to 12 years old to screen for early signs of CCALD. If symptoms occur suggestive of cerebral ALD (for instance declining school performance) the MRI should be performed at the earliest available opportunity, but it is our experience that the detection of brain MRI abnormalities precedes any detectable cognitive dysfunction by at least 6 months to 1 year. After the age of 12 years, the incidence of CCALD decreases, but an MRI scan must be performed yearly or earlier if new symptoms occur. It is important to detect cerebral ALD as early as possible, preferably in the asymptomatic stage with only moderate radiological abnormalities to discuss the possibility to perform allogeneic HCT. Accordingly, if a brain MRI shows abnormalities, even very limited such as an increased signal intensity on T2 or FLAIR sequences in the splenium or genu of the corpus callosum, brain MRI must be repeated within 3 months to evaluate disease progression and in particular to identify the presence of gadolinium rim enhancement of lesions. Because the disease can be very rapidly progressive, it is strongly advised to discuss the possibility of allogeneic HCT as soon as brain MRI abnormalities typical of cerebral ALD are detected. After a successful transplant, the lesions on MRI stabilize and even regress. Treatment results are better the earlier treatment is started [82].

For adult men with or without signs of AMN, we advise evaluation by a neurologist yearly or bi-annually to screen for symptoms of AMN and to administer symptomatic treatment if necessary (for instance, medication against spasticity). Referral to a rehabilitation physician and urologist will often become necessary.

Adult men can develop cerebral ALD and in our centers, we offer an MRI of the brain every single year [30,45]. There is no proven treatment for cerebral ALD in adults. It seems likely that allogeneic HCT is also effective in adults with early stage cerebral ALD, but there are no published studies or cases describing this treatment. We tend to consider allogeneic HCT in an adult patient with early stage cerebral ALD, after carefully counseling the patient about the lack of evidence for the treatment and the risk of the procedure which is significantly higher than in boys. Whereas the onset of demyelinating lesions involving the corpus callosum and adjacent parieto-occipital or frontal white matter leaves no doubt about the onset of cerebral ALD, the situation is different when there are only slightly increased signal abnormalities in the pyramidal tracts of AMN patients that gradually become more intense and involve the white matter of the centrum semiovale. This can herald the onset of cerebral ALD, but can also reflect Wallerian degeneration in severe AMN.

For AMN there is no effective disease modifying therapy available yet. Although Lorenzo’s oil (LO) had great promise, several open-label trials have shown that the disease progresses even when plasma VLCFA are normalized by LO treatment [84,85]. A large randomized placebo-controlled clinical trial was designed to provide a definitive answer, but was unfortunately aborted before completion by the safety monitoring board because of presumed side effects of the placebo treatment. There is also a retrospective study suggesting that if presymptomatic boys are started on LO, it may delay the onset of neurological symptoms [86]. We consider the scientific evidence to support the efficacy of LO weak, and do not offer this treatment to our patients. Regular follow-up in AMN remains important, however, mainly to provide symptomatic treatment.

Lovastatin also lowered plasma VLCFA [87], but a placebo-controlled trial revealed that lovastatin did not have an effect on the C26:0 levels in peripheral blood lymphocytes and erythrocytes nor on the VLCFA content of the low-density lipoprotein fraction [88].

More research and new treatments strategies are desperately needed, especially for those affected by AMN, which is relentlessly progressive and causes severe disability. Antioxidants reduce markers for oxidative stress and axonal degeneration in the spinal cord of *Abcd1* knockout mice [89]. Based on this observation a clinical trial with anti-oxidants in X-ALD is ongoing in Spain.

### Women with X-ALD

Women with X-ALD should be evaluated for the development of neurologic symptoms. Since women with X-ALD very rarely develop adrenocortical insufficiency or cerebral involvement, periodic evaluation of adrenocortical function and brain MRI is not mandatory [37]. Greater awareness among physicians that women can develop neurologic symptoms is important for counseling but also to prevent unnecessary diagnostic tests and erroneous diagnosis. We know of cases of women with X-ALD who underwent cervical laminectomy for presumed cervical spondylogenic myelopathy. For symptomatic women with X-ALD, we advise (as for men with AMN) a yearly evaluation by a neurologist to discuss the indication of rehabilitation, the referral to an urologist and treatment of spasticity and neuropathic pain.

## Prognosis

It is debatable whether men with X-ALD can remain really asymptomatic for life. It seems probable that there is only presymptomatic X-ALD in men. Although there is no large prospective cohort study to accurately determine the natural history of the disease, several general observations can be made. It is likely that all patients with X-ALD if they survive into adulthood eventually develop myelopathy, i.e. AMN. Usually, symptoms and signs occur from the 3^rd^ decade of life, but much earlier, or much later, is possible. The severity and progression cannot be predicted for individual patients. There is marked variability ranging from men with X-ALD that are wheelchair bound by the age of 25 and others who are able to walk with a cane while in their seventies. This is important when counseling patients: symptoms will occur, but only time will tell how severely affected an individual will be.

Not every boy or man with X-ALD will develop cerebral ALD. About 35-40% of boys with an *ABCD1* mutation will develop CCALD before reaching adulthood. It can still not be predicted who will develop this devastating manifestation of the disease. Previously it was believed that after reaching adulthood this complication was very rare. However, it is now well established that at least 20% of adult males with the AMN phenotype will develop cerebral demyelination later in life [45]. If this occurs, these patients have the same poor prognosis as boys with inflammatory cerebral ALD.

It is well documented that women develop symptoms that resemble AMN [46], but there is no prospective study with adequate numbers to really estimate what percentage of women become symptomatic. We recently completed a large prospective cohort study to describe the symptomatology of X-ALD in women (Engelen et al, manuscript in preparation).

## Unresolved questions and future research

For the majority of patients with X-ALD there is currently no curative or preventive treatment. However, several promising new approaches will hopefully come to fruition in the future. For example, it has been demonstrated in X-ALD cells that small interfering RNA (siRNA)-mediated inhibition of ELOVL1 reduces VLCFA synthesis and levels [6]. Compounds that can inhibit ELOVL1 are therefore interesting candidates for new preventive treatments. We demonstrated that bezafibrate which is both an effective, safe and well-tolerated compound lowers the levels of VLCFA in X-ALD cells by inhibiting VLCFA synthesis (Engelen et al., in press http://dx.doi.org/http://10.1007/s10545-012-9471-4). A clinical trial to evaluate its *in vivo* efficacy in X-ALD patients is ongoing.

As mentioned before, it is likely that several modifier genes play a role in the onset and severity of AMN, the onset of cerebral ALD and the progression to the rapidly progressive neuroinflammatory stage. However, so far no major modifier genes have been identified that could lead to the development of a genetic test to predict phenotype and disease evolution. The eventual phenotype of X-ALD in an individual will most likely be determined by the combination of several epigenetic and environmental modifiers, most of which have not been identified. Much research is currently focused on identifying these modifiers to better predict clinical outcome in individual patients.

An important issue is the systematic characterization of disease manifestations in women with X-ALD. Clinical research has always focused on boys and men and women were often considered “carriers” with no or minimal symptoms. For adequate counseling this information is important and should be available.

For clinicians caring for patients with AMN it is well known that cerebral demyelination can occur. It is important to systematically study if an allogeneic HCT can be successful in these cases and even arrest the progression of myelopathy.

## Conclusions

Pediatricians, endocrinologists, neurologists and psychiatrists may encounter X-ALD which is a relatively common metabolic disorder in their practice. The disorder is associated with severe morbidity and mortality in the majority of affected patients. Recognition of X-ALD is highly important, since in some cases treatment is available, such as allogeneic HCT in the early stage of CCALD and endocrine replacement therapy for adrenocortical insufficiency. Furthermore, prenatal testing to prevent unnecessary new cases of this devastating disease is available.

## Abbreviations

ACALD: Adult cerebral adrenoleukodystrophy; ACOX1: Acyl-CoA oxidase 1; ACTH: Adrenocorticotropic hormone; AdolCALD: Adolescent cerebral adrenoleukodystrophy; ALDP: Adrenoleukodystrophy protein; AMN: Adrenomyeloneuropathy; CCALD: Childhood cerebral adrenoleukodystrophy), DBP, D-bifunctional protein; FLAIR: Fluid attenuated inversion recovery); HCT: Hematopoietic stem cell transplantation; LO: Lorenzo’s oil; MRI: Magnetic resonance imaging; VLCFA: Very long-chain fatty acids; X-ALD: X-linked adrenoleukodystrophy.

## Competing interests

The authors’ declared that they have no competing interest.

## Authors’ contributions

All authors were involved in the conception and writing of the manuscript. All authors read and approved the final manuscript.
